# The use of back propagation neural networks and ^18^F-Florbetapir PET for early detection of Alzheimer’s disease using Alzheimer’s Disease Neuroimaging Initiative database

**DOI:** 10.1371/journal.pone.0226577

**Published:** 2019-12-26

**Authors:** Ilker Ozsahin, Boran Sekeroglu, Greta S. P. Mok

**Affiliations:** 1 Biomedical Imaging Laboratory, Department of Electrical and Computer Engineering, Faculty of Science and Technology, University of Macau, Macau SAR, China; 2 Department of Biomedical Engineering, Faculty of Engineering, Near East University, Nicosia, Turkey; 3 Department of Information Systems Engineering, Near East University, Nicosia, Turkey; 4 Faculty of Health Sciences, University of Macau, Macau SAR, China; University of South Florida, UNITED STATES

## Abstract

Amyloid beta (Aβ) plaques aggregation is considered as the “start” of the degenerative process that manifests years before the clinical symptoms appear in Alzheimer’s Disease (AD). The aim of this study is to use back propagation neural networks (BPNNs) in ^18^F-florbetapir PET data for automated classification of four patient groups including AD, late mild cognitive impairment (LMCI), early mild cognitive impairment (EMCI), and significant memory concern (SMC), versus normal control (NC) for early AD detection. Five hundred images for AD, LMCI, EMCI, SMC, and NC, i.e., 100 images for each group, were used from the Alzheimer’s Disease Neuroimaging Initiative (ADNI) database. The results showed that the automated classification of NC/AD produced a high accuracy of 87.9%, while the results for the prodromal stages of the disease were 66.4%, 60.0%, and 52.9% for NC/LCMI, NC/EMCI and NC/SMC, respectively. The proposed method together with the image preparation steps can be used for early AD detection and classification with high accuracy using Aβ PET dataset.

## 1 Introduction

Alzheimer’s disease (AD) is the most common type of neurodegenerative disease that accounts for approximately 60% to 80% of dementia cases [1], leading to an irreversible and progressive mental deterioration as a result of neuronal death in the brain. Currently, there are approximately 47 million people affected by the disease around the globe [2] and it is expected to increase dramatically to over 100 million by 2050 [3]. There are presently no clinically proven treatments to avoid or stop the progression of the disease, as they only target the symptoms of AD rather than its cause [4]. The main underlying reason might be the late diagnosis from common behavioral diagnostic approaches, leading to late intervention where irreversible brain damage has already happened. Thus, given that AD can be detected at earlier stages, clinical treatments would have a greater impact on disease progression, highlighting the importance of developing early and effective detection methods.

AD is neuro-pathologically characterized by the presence of amyloid plaques (amyloid beta (Aβ) protein) and neurofibrillary tangles (NFTs) (hyper-phosphorylated tau protein), along with deficits in certain neurotransmitter systems [5]. The “amyloid cascade hypothesis” is accepted as the “start” of the neurodegenerative process, which states that Aβ aggregation, either due to overproduction of Aβ or failing to clear this peptide, leads to plaque formation and deposition as well as NFT production, and eventually neuronal death that gives rise to the clinical manifestations of the disease [6]. To this end, Aβ can be used as a core biomarker of AD as an early detection method that may begin to appear years before initial symptoms surface [7].

Neuroimaging based methods, such as magnetic resonance imaging (MRI) and positron emission tomography (PET) imaging, have been used to diagnose AD at its early stages and assess disease progression [8] by providing an in vivo measure of brain changes linked with the disorder [9]. PET imaging is a functional imaging technique that provides unique information with great sensitivity in detecting the presence of early events and tracking brain alterations over time [10, 11]. Thus, it is considered as a promising candidate for assessing the Aβ load in vivo [8] for early detection of the disease [5]. Various agents are being used to image AD pathogenesis using the Aβ biomarker, including the commonly used radiotracers of ^11^C-labelled Pittsburgh Compound-B (^11^C-PIB) [12] and ^18^F-florbetapir (^18^F-AV45) [13].

Extraction and classification of clinical data for such a heterogeneous and multivariate disease as AD has always been challenging [14]. Literature emphasizes the role of computer-aided diagnostic techniques with the goal of gaining a more in-depth understanding of the disease process and diagnostic classification [15]. To this end, machine learning techniques can be employed to capture complex patterns of data [16] for an early and accurate prediction of the disease, and differentiate AD patients not only from healthy individuals but also from individuals suffering from mild cognitive impairment (MCI) (both early (EMCI) and late (LMCI) stages) and significant memory concern (SMC). MCI is the prodromal stage of AD that has the potential to convert to AD with different rates of cognitive decline or remain the same with no further disease progression [17], while SMC individuals are cognitively normal with significant memory concerns [18]. It is therefore a research interest to distinguish the preclinical/prodromal stages of AD [19], allowing for revised diagnosis criteria and early interventions. The clinical diagnostic criteria for AD were revised for the first time in 2011 by the National Institutes on Aging and the Alzheimer’s Association, and research guidelines for earlier stages of the disease describe the earliest preclinical stages of the disease, mild cognitive impairment, and dementia due to Alzheimer’s pathology to reflect a better understanding of the disease [20].

Machine learning deals with the models to teach some data to computers or machines to make decisions over untrained data that traditional algorithms are unable to do. They integrate computers’ more flexible decision-making boundaries for different kinds of problems such as classification and regression etc. This integration can be based on mathematical improvements of statistics or other disciplines such as neural networks and deep learning.

An artificial neural network (ANN) is a type of machine learning algorithm that allows systems to learn from data and improve from experience without any human intervention. It uses training sets of real-world data to deduce models with a greater accuracy than human intervened models by stimulating the neural structure of the brain. It is proven to perform better in extracting the biomarkers of heterogeneous datasets as in AD where the data volume and variety are huge [21].

In classification and prediction problems, it can be said that the type of data is important for model selection. Neural networks are preferred if the data is not linearly separable even though they are time consuming during the training stage. The amount of data is also important because large amount of data and output classes may need deep-learning approaches to provide effective learning; however, this increases the computational cost. For this reason, image preprocessing steps are still an effective way to prepare efficient data for shallow neural networks or other machine learning models.

Deep learning has high accuracy and reliability for classification of disease stages, and thus making effective diagnosis. However, training phases of deep learning methods need considerable time. Image processing techniques are used to reduce the amount of data to the neural network to minimize training and testing time while increasing the performance and accuracy for limited class problems [22,23,24].

In the current study, we propose the use of back propagation neural networks (BPNNs) for the automated classification of 4 groups of patients including AD, LMCI, EMCI, SMC versus normal controls (NC) on ^18^F-florbetapir PET images from the Alzheimer’s Disease Neuroimaging Initiative (ADNI) database, with 100 images for each group. The proposed system consists of two stages: image preparation and artificial neural networks. Basic and effective image processing techniques are applied in order to prepare data for the shallow BPNN.

## 2 Material and methods

### 2.1 Datasets

The datasets used in the current study were downloaded from the open Alzheimer’s Disease Neuroimaging Initiative (ADNI) database (adni.loni.usc.edu). The ADNI is a public-private partnership, led by Principal Investigator Michael W. Weiner, MD., and was launched in 2003. The goal of ADNI is to test whether serial magnetic resonance imaging (MRI), positron emission tomography (PET), other biological markers, and clinical and neuropsychological assessment can be combined to measure the progression of MCI and AD at its early stage by providing very large clinical and imaging datasets. For up-to-date information, see www.adni-info.org. Informed consent was obtained for all subjects, and the study was approved by the relevant institutional review board at each data acquisition site (for up-to-date information, see http://adni.loni.usc.edu/wp-content/themes/freshnews-dev-v2/documents/policy/ADNI_Acknowledgement_List%205-29-18.pdf). All methods were performed in accordance with the relevant guidelines and regulations.

For this study, a total of 500 images, i.e., 100 images for each group, were used for five groups denoted as AD, LMCI, EMCI, SMC, and NC. Therefore, each image belongs to a different subject. For NC group, data were selected from a combination of ADNI1/GO and ADNI2 while for AD, LMCI, EMCI, and SMC groups, all data were selected from ADNI2. However, we randomly selected 100 baseline images for each group (except AD) among the larger population. Subject ID numbers are presented in the supplement file for reproducibility purpose. Table 1 shows the phenotypic data for each group.

**Table 1:** Phenotypic data (MMSE = Mini-Mental State Exam, APOE ε4: ε4 allele of apolipoprotein E.

State	Avg. Age (SD)	Avg. MMSE (SD)	Gender	APOE ε4 carriers
AD	74.8 (8.2)	18.2 (3.9)	58 M, 42 F	66
LMCI	75.6 (7.9)	25.3 (3.8)	63 M, 37 F	51
EMCI	74.4 (8.1)	27.7 (1.8)	58 M, 42 F	54
SMC	73.4 (5.7)	28.8 (1.4)	43 M, 57 F	31
NC	77.3 (7.2)	29.2 (1.1)	42 M, 58 F	34

In the dataset we used, 66% of the AD patients carried at least one APOE ε4, while this rate was 51%, 54%, 31%, and 34% for the LMCI, EMCI, SMC, and NC groups, respectively (Table 1). On the other hand, people with MCI might be converted to AD or remain stable, while some of them even get better and convert to NC. In the dataset we used, 59% of LMCI patients converted to AD (LMCI-converters), 38% remained stable (LMCI-non-converters) and 3% converted to NC. 59.3% of LMCI-converters carried APOE ε4. For EMCI patients, 16% of them converted to AD (EMCI-converters), while 74% remained stable (EMCI-non-converters) and 10% converted to NC. 68.8% of LMCI-converters carried APOE ε4. The conversion rate for SMC patients was 1%. The details are presented in Table 2.

**Table 2:** Number of MCI-converters and MCI-non-converters, and the number of their APOE ε4 carriers.

	LCMI	EMCI
APOE ε4 carriers / MCI-converter	35/59 (59.3%)	11/16 (68.8%)
APOE ε4 carriers / MCI-non-converter	13/38 (34.2%)	37/74 (50.0%)
APOE ε4 carriers / Converted-to-NC	2/3 (66.7%)	6/10 (60.0%)

**Fig 1:**
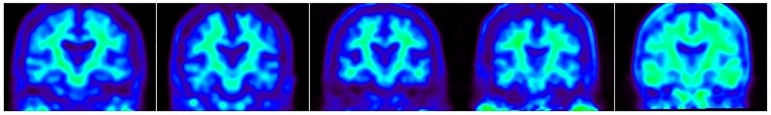
Sample ^18^F-florbetapir PET images. From left to right: 66-year-old normal control (MMSE, 30), 73-year-old individual with SMC (MMSE, 29), 84-year-old subject with EMCI (MMSE 27), 64-year-old subject with LMCI (MMSE 26), 71-year-old patient with AD (MMSE 19). All images were scaled to the same maximum. PET images show clear differences in the cortical region between AD and NC, but not LMCI, EMCI, SMC vs. NC visually.

Subjects were injected with 10 mCi ±10% ^18^F-florbetapir and acquisition started at 50–90 min post injection. A dynamic 3D scan consisting of four 5-min frames, i.e., a total of 20 min, was performed. Fully 3D iterative image reconstruction was performed with 4 iterations and 20 subsets. Fig 1 shows the sample images from each group.

### 2.2 Preprocessing

The goal of the preprocessing steps is to make PET data more uniform and to increase the similarities of PET images if they are obtained by different scanners. These will provide consistency and more efficient data analysis. The preprocessing was performed by ADNI; however, further image processing steps that we implemented are described in the following sections. The details of ADNI preprocessing can be summarized as follows: co-registration of frames, averaging the frames, standardization and resolution.

#### Co-registration

PET data from scanners in all sites are downloaded for quality control from the University of Michigan. Firstly, the data are converted to a standard file format. Separate frames are extracted from the image file for registration purposes. Each extracted frame (4 frames in total) is co-registered to the first extracted frame of the raw image file. The base frame image and the four co-registered frames are recombined into a co-registered dynamic image set. These image sets have the same image size and voxel dimensions and remain in the same spatial orientation as the original PET image data.

#### Averaging

This type of processed image set is essentially generated by averaging the four 5-minute frames of the co-registered image set described above. A single 20 min PET image set is created at the end of this process.

#### Standardization

Each subject’s co-registered and averaged image from the raw data is then reoriented into a standardized size. This image grid is oriented such that the anterior-posterior axis of the subject is parallel to the anterior commissure—posterior commissure line. This standardized image then serves as a reference image for all PET scans on that subject. The individual frames from each PET scan are co-registered to this baseline reference image.

#### Resolution

Each image set is filtered with a scanner-specific filter function to produce images of a uniform isotropic resolution of 8 mm FWHM. The specific filter functions were determined from the Hoffman phantom PET scans that were acquired during the certification process. More detailed information about each step can be found at http://adni.loni.usc.edu/methods/pet-analysis/pre-processing/.

### 2.3 Image processing

**Fig 2:**
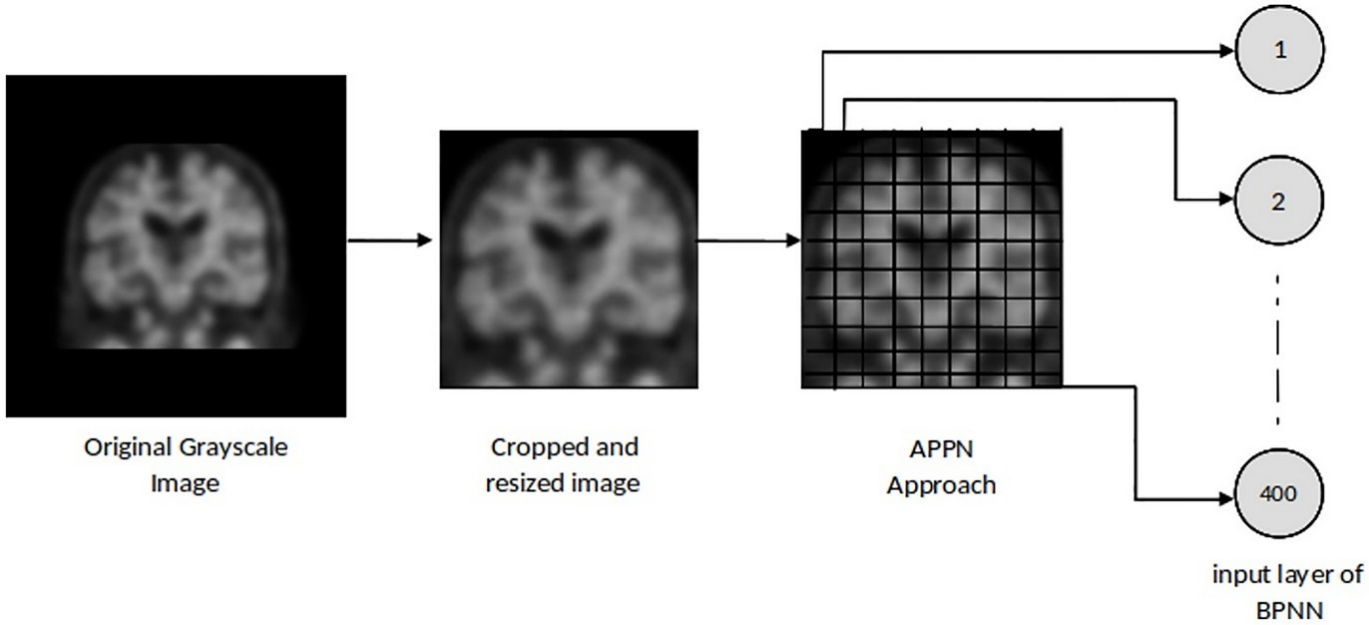
The steps of image preparation phase.

Our image processing method includes the preparation and minimization of data to minimize the training time and to optimize the recognition skills of neural networks. In the initial step of image processing, images are automatically cropped to remove black frames from the region of interest, which increases the computational cost during the training and generalization with zero values. Then, cropped images are resized to 200×200 pixels in order to apply the Average Pixel Per Node (APPN) approach. The APPN is based on dividing images into predefined segments and calculating the average of the gray level values for each segment. Thus, reduced and statistically measured inputs are provided for the neural network. In this paper, a 10×10 segment size was used for APPN and in total, 400 normalized input values were fed to the neural network. Fig 2 shows the steps of the image preparation phase in detail.

### 2.4 Back Propagation Neural Networks (BPNNs)

Back Propagation (BP) is one of the most widely used and effective supervised learning algorithms in ANNs. Ease of implementation and fast convergence make BP popular for researchers requiring shallow neural networks. BP has been used to solve real-life classification problems with superior results. Therefore, it can be used for any classification problems or applications using medical data in order to simulate and improve the human recognition skills.

**Fig 3:**
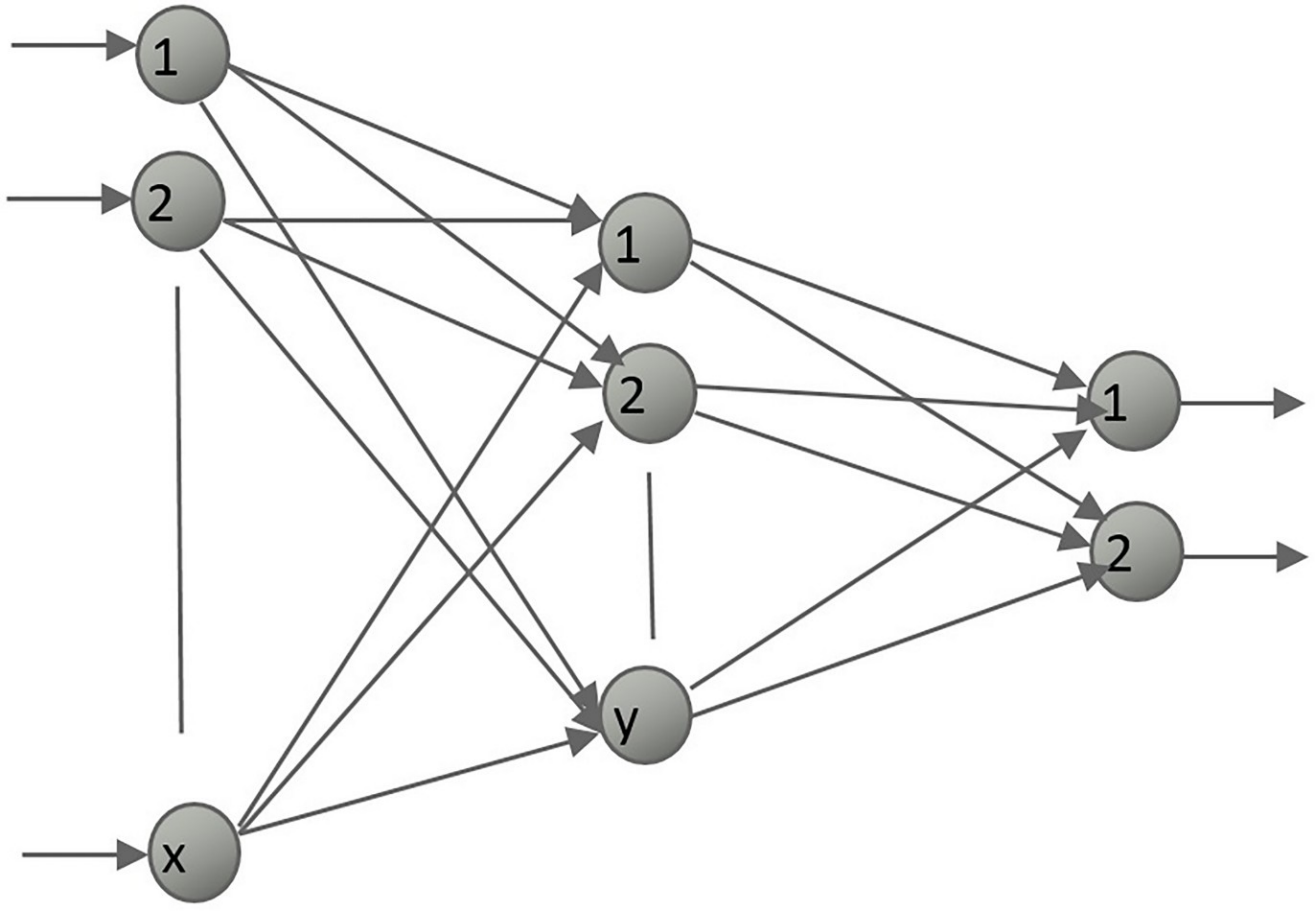
General topology of 3-layered back propagation neural network for x inputs, y hidden neurons and 2 outputs.

Normalized values are sent to the input layer of the neural network with or without preprocessing and the total potential of each neuron of the hidden layer is calculated by using these input values and initially randomized weights. Then, the output of each neuron is calculated using the activation function. These steps are repeated in the next layer, which is the output layer for a 3-layered neural network. It uses gradients to update the weights and biases during the training by propagating the error signal to the back. Learning and momentum rates are used to increase the learning speed and to avoid the local minima during the convergence. All these feed forward and feedback calculations are repeated until the stopping criteria, generally Root Mean Square (RMS) error, is reached. The general topology of the 3-layered BPNN is shown in Fig 3.

There are different types of neuron activation functions such as Logistic Sigmoid which produces smooth slope between 0 and 1, Tangent Sigmoid function which produces output between -1 and 1, Hard Activation Function which produces only 0 or 1, and Rectified Linear Unit (RELU) function which produces linear slope between 0 and 1. Tangent Sigmoid and Hard Activation is not considered for this research because of the differences of outputs but Logistic Sigmoid and ReLU functions are used. Also, number of hidden neurons, learning rate and momentum factors are other parameters that affect the accuracy both in training and testing phases. However, there is not an exact rule that gives an optimum activation function or other parameter values. Thus, several experiments (training and testing of neural network) were performed with different parameter values and with different activation functions to determine the optimum neural network structure both in training and testing. In this research, the hold-out method is considered to build the initial model and to obtain results efficiently in terms of time. It is one of the effective ways to train neural network while it only consists one train-test step and mainly depends on randomly selected training patterns.

BPNN is used for training 30% of the total images for each class that consists of 100 images. The hidden neuron number is determined as 45 in the hidden layer after performing several experiments. The momentum rate, learning rate and minimum RMS error are set to 0.90, 0.00079 and 0.003, respectively, for all experiments. Increments or decrements of the momentum rate and learning rate do not affect the testing performance of the system, but cause late or no convergence during training. The minimum RMS error is set to 0.003 by performing several experiments in interval of 0.0002 to 0.003. The maximum and minimum values of considered RMS error are 0.005 and 0.001. The hidden neuron number is another challenge in neural networks and experiments are performed by considering 20 to 100 neurons with an interval of 5. After all experiments, optimum results are obtained based on a hidden neuron number of 45, momentum rate of 0.90, learning rate of 0.00079 and a minimum RMS error of 0.003 for all experiments.

Table 3 shows the final training parameters of BPNN. BPNN is implemented in C language and experiments are performed on Windows 10 OS, with 8 GB RAM and Intel^®^ Core(TM) i5-3230M 2.60 GHz CPU.

**Table 3:** Common BPNN parameters.

Parameters	Value
Input Neuron Number	400
Hidden Neuron Number	45
Output Neuron Number	2
Learning Rate	0.00079
Momentum Rate	0.90
Minimum RMS Error	0.0030
Activation Function	Sigmoid
Bias Weights	Used

## 3 Results

The NC group was compared with other four groups, namely AD, LCMI, EMCI, and SMC. The generalization (test) was performed using 70% of the untrained data of each class for each experiment. In the first experiment, randomly selected NC and AD images were used to train the BPNN and the convergence of neural network was completed after 2837 iterations and in 70.89 seconds. Sensitivity, specificity and accuracy in this experiment are measured as 92.4%, 84.3%, and 87.9%, respectively. In the second experiment, NC and LMCI images were considered. The convergence completed after 3680 iterations and in 91.85 seconds. Sensitivity, specificity and accuracy of 62.9%, 70.0% and 66.4% were achieved, respectively. In the third experiment, NC and EMCI images were used for training. The learning of BPNN was completed after 3886 iterations and in 96.92 seconds. Sensitivity of 60.0%, specificity of 60.0% and accuracy of 60.0% were obtained. Finally, in the last experiment, NC and SMC images were used to train the BPNN. Training was completed after 4884 iterations and in 122.67 seconds. Sensitivity, specificity and accuracy values of 60.0%, 45.7% and 52.9% respectively were obtained for the data to be tested. A training performance of 100% was obtained for each experiment. Table 4 summarizes the results for each experiment.

**Table 4:** Results for all experiments.

Experiment	Sensitivity (%)	Specificity (%)	Accuracy (%)
NC/AD	92.4	84.3	87.9
NC/LCMI	62.9	70.0	66.4
NC/EMCI	60.0	60.0	60.0
NC/SMC	60.0	45.7	52.9

The confusion matrix showing true positive, true negative, false positive and false negative values is presented in Table 5.

**Table 5:** Results of prediction condition.

Binary classification	NC/AD	NC/LMCI	NC/EMCI	NC/SMC
True positive	64/70	44/70	42/70	42/70
True negative	59/70	49/70	42/70	32/70
False positive	11/70	21/70	28/70	38/70
False negative	6/70	26/70	28/70	28/70

In this study, we used 100 images for each group and the number of converters/non-converters is too low to make further discrimination between them. Also considering that this number will be further decreased after using some of them for training, the results will not be accurate with the current dataset. However, we considered the information on conversion and genotype (Table 2) to see how BPNN performed when such information is provided. The results showed that a total of 19 of the 24 subjects (79.2%) who were both APOE ε4 carriers and LMCI-converters were classified as LMCI with our method, which is very promising to proceed with the prediction studies in the further studies.

## 4 Discussion

The classification of AD using ANNs has been studied for many years and reached high accuracy. However, although many studies have used MRI data to assess brain atrophy, MRI is not able to detect the hallmarks of AD for MCI patients who are at risk of AD. This is because MRI is only useful to assess progressive cerebral atrophy that is a later stage after Aβ aggregation and tau mediated neuronal dysfunction in the proposed AD pathological cascade [25].

To date, almost all studies with PET for AD have been done on ^18^F-FDG, which mainly shows synaptic activity/dysfunction, and in those studies, mostly NC/AD and NC/MCI classifications have been assessed [8, 26–29]. Since the molecular pathology of AD can be visualized with amyloid PET imaging, in our study, we are able to use ANN method to classify AD, LMCI, EMCI, and SMC versus NC, in which some of these groups include subjects in prodromal (MCI) and preclinical (SMC) stages. Table 6 summarizes the results of different reports on classification of AD and some of its stages with different neural network algorithms on the ^18^F-florbetapir PET dataset. The results show that our method based on ^18^F-florbetapir PET data can be used successfully in the classification and early detection of AD.

**Table 6:** Summary of recent studies on AD classification accuracy. (RF: Random Forest; SRC: Sparse Representation-based Classification; SCDDL: Supervised within-Class-similarity Discriminative Dictionary Learning).

Accuracy (%)					Method	Agent	References
NC/AD	NC/MCI	NC/LMCI	NC/EMCI	NC/SMC			
87.9	N/A	55.7	59.7	N/A	RF	Florbetapir	[29]
83.9	70.5	N/A	N/A	N/A	SRC	Florbetapir	[30]
85.6	70.1	N/A	N/A	N/A	SCDDL	Florbetapir	[31]
87.9	N/A	66.4	60.0	52.9	BPNN	Florbetapir	This study

We achieved high accuracy for NC/AD classification, while the NC/LMCI classification results were promising, followed by NC/EMCI and NC/SMC. The low classification accuracy for some of the non-AD groups could be attributed to that these groups might include both biomarker-positive and biomarker-negative individuals. The presence of cerebral amyloid pathology in MCI, SMC, and even NC persons varies from 10% to 80% depending on the risk factors for AD [32]. Therefore, non-AD individuals might be amyloid-positive and might or might not convert to AD [33]. Another study showed that Aβ aggregation can be positive in non-AD subjects due to the fact that 20%-40% of cognitively normal or non-AD individuals, and especially these individuals are older and carry APOE ε4 [20]. Therefore, some of the NC group patients are probably Aβ-positive. Thus, we believe the main factor behind the low classification accuracy for the prodromal stages is because the patients classified as MCI (either LMCI or EMCI) and SMC might be either amyloid-positive or negative, together with the fact that 20%-40% of the NC group might be Aβ-positive, which directly affects the classification accuracy.

The conventional analysis has proved to agree with the autopsy to some degree. Interpretation of PET data in the setting AD is usually a manual approach using visual qualitative reading or a reference region based quantitative analysis. A manual approach can be prone to interpretation errors and strongly depends on the experience and training of the reading physician. Furthermore, it requires highly experienced board certificated nuclear medicine and radiology physicians (usually 2 to 5 readers) to interpret the amyloid beta images. Also, there are factors affecting the quantitative analysis such as selecting the reference region from cerebellum or white matter. Quantitative regional analysis of amyloid PET data is a time-consuming and complex process which also requires MRI coregistration. Region of interest (ROI) analyses have been the mainstream to investigate the changes in many studies related with AD. However, studies utilizing the ROI analyses exclusively depend on previous experience on ROI selection. In addition, they do not consider the regions outside of the ROIs which might include further information. Artificial intelligence offers an efficient methodology and objective classification systems for analyzing high-dimensional and large dataset. It can learn complex patterns and changes cannot be detected visually. Single pixel in an image may change the class of image but it cannot be perceived by human beings. BPNN uses normalized inputs of images and their corresponding weights to identify this change. This ability is obtained during the training which consists of error minimization and weights adjustments using gradient-descent algorithm.

Finally, but most importantly, the National Institute on Aging at the National Institutes of Health and the Alzheimer’s Association recently published a research framework for the biological definition of AD [34]. According to this newly revised definition, "An individual with biomarker evidence of Aβ deposition (e.g. abnormal amyloid PET scan) with an evidence of pathologic tau biomarker would be assigned the label *Alzheimer’s disease*". Therefore, imaging the tau pathology is as important as imaging the biomarker Aβ in the early detection of AD. On the other hand, neurodegenerative injury biomarkers and cognitive symptoms are not considered as specific to AD as opposed to Aβ and pathologic tau biomarkers, which are specific neuropathological changes that define AD. In addition, in the three cognitive stages, namely cognitively unimpaired, MCI, and dementia, syndromes are combined with biomarkers for a broader definition. Therefore, we believe that further studies should be designed according to these new broader definitions and the staging of the disease.

By considering all the factors mentioned above, in our further studies, we will use additional datasets including other imaging agents such as FDG for brain glucose utilization and flortaucipir for tau protein imaging as supportive inputs to increase the accuracy of each classification. Age, APOE genotype, education, and sex should also be considered for a more accurate classification. Moreover, the new dataset including risk factors could be useful for prediction studies to foresee whether an MCI patient will convert to AD in future. In fact, when considering the APOE genotype and the conversion information of LMCI patients, ~80% of the subjects who are both APOE ε4 carriers and LMCI-converters were classified as LMCI with our method.

In this study, we showed that traditional BPNN can effectively be used for the classification of AD with image preprocessing, which minimizes training time and increases the testing performance of the system. The obtained results are superior to other recent researches and this makes our system more efficient in real-life implementation with higher accuracy. With additional datasets on a different biomarker like pathologic tau, our method can be used for higher classification accuracy and prediction studies.

## Supporting information

S1 Data (XLSX)
